# Rescue of Long-Term Spatial Memory by 7,8-Dihydroxyflavone in Mice with Reduced Oligodendrogenesis

**DOI:** 10.1523/ENEURO.0498-22.2023

**Published:** 2023-05-04

**Authors:** Florence Rawlings-Mortimer, Heidi Johansen-Berg

**Affiliations:** Wellcome Centre for Integrative Neuroimaging, Nuffield Department of Clinical Neurosciences, University of Oxford, Oxford OX3 9DU, United Kingdom

## Abstract

Oligodendrogenesis is the process by which new oligodendrocytes are produced in the CNS. Oligodendrocytes form myelin, which has a vital role in neural signal transmission and integration. Here we tested mice with reduced adult oligodendrogenesis in the Morris water maze, a test of spatial learning. These mice were found to have impaired long-term (28 d) spatial memory. However, when 7,8-dihydroxyflavone (7,8-DHF) was administered immediately after each training session, their long-term spatial memory impairment was rescued. An increase in the number of newly formed oligodendrocytes in the corpus callosum was also observed. 7,8-DHF has previously been shown to improve spatial memory in animal models of Alzheimer’s disease, post-traumatic stress disorder, Wolfram syndrome and Down syndrome, as well as in normal aging. Understanding the underlying mechanisms of the effect of this drug on spatial memory is therefore helpful in assessing it for clinical relevance and development.

## Significance Statement

7,8-dihydroxyflavone (7,8-DHF) is a drug that has been shown to improve the symptoms of numerous brain disorders including Alzheimer’s disease and post-traumatic stress disorder in mouse models. It is therefore of great interest clinically to understand the impact of this drug on the brain and assess behavioral changes over longer time periods. Here, we show that 7,8-DHF improves spatial memory 1 month after administration in mice with reduced numbers of new oligodendrocytes in adulthood. We also found an increase of newly formed oligodendrocytes in the corpus callosum, providing insights into the long-term effects of this drug.

## Introduction

Oligodendrocytes are the myelin-producing cells in the CNS. Myelin has many important roles including facilitation of neuronal signaling. Oligodendrocytes develop from oligodendrocyte precursor cells (OPCs) with myelin regulatory factor (MyRF) thought to play a key role in this process (Emery et al., 2009). Mice with selective and conditional deletion of *Myrf* in OPCs were developed (Emery et al., 2009; McKenzie et al., 2014). These MyRF^−/−^ mice have impaired oligodendrogenesis and reduced numbers of newly formed mature oligodendrocytes. They have previously been shown to have impairment in long-term spatial and contextual fear memory consolidation (Pan et al., 2020; Steadman et al., 2020) and reduced ability to undergo remyelination (Duncan et al., 2017).

There is growing evidence that brain-derived neurotrophic factor (BDNF) acting via tyrosine kinase B (TrkB) receptors may have a pro-myelinating influence in the CNS. BDNF was found to enhance myelination *in vitro* via oligodendrocyte TrkB receptors (Xiao et al., 2010). BDNF knock-out (KO) mice (Korte et al., 1995) have reduced expression of myelin basic protein and proteolipid protein as well as proportionally fewer myelinated axons in the optic nerve (Cellerino et al., 1997; Djalali et al., 2005; Vondran et al., 2010). There is also evidence that BDNF may play a role in spatial memory. Aged mice that lack the intracellular glucocorticoid-regenerating enzyme 11β-hydroxysteroid dehydrogenase type 1 were found to have increased BDNF mRNA levels and improved spatial memory formation (Yau et al., 2007; Caughey et al., 2017).

7,8-dihydroxyflavone (7,8-DHF) is a low-molecular-weight compound that is thought to be a TrkB receptor agonist (Jang et al., 2010; Massa et al., 2010), although recent research suggests that it may work through alternative mechanisms (Pankiewicz et al., 2021). Previous research has shown improved short-term Morris water maze (MWM) performance following 7,8-DHF administration for example, in three different Alzheimer’s disease (AD) mouse models (Tg2576, 5XFAD, and CaM/Tet-DT_A_; Castello et al., 2014; Zhang et al., 2014; Gao et al., 2016). 7,8-DHF was found to improve short-term MWM memory in male rats that had previously been subjected to immobilization stress [used to elicit post-traumatic stress disorder (PTSD) symptoms; Andero et al., 2012], a Wolfram syndrome rat model (Seppa et al., 2021), a Down syndrome mouse model (Stagni et al., 2017), and aged rats (Zeng et al., 2012). However, the impact of 7,8-DHF on long-term spatial memory and oligodendrogenesis has not been investigated.

We investigated the effect of administering 7,8-DHF during the MWM training period on spatial memory 28 d later in MyRF^−/−^ mice. We also evaluated the outcome of 7,8-DHF on the numbers of newly formed oligodendrocytes in the corpus callosum of these mice. To assess potential side effects of 7,8-DHF on adult neurogenesis, we tested for changes in the number of newly formed neurons in the hippocampus of the MyRF^−/−^ mice.

## Materials and Methods

### Experimental animals

The PDGRFα-CreERT2:Rosa26R-eYFP:Myrf mouse line was used as previously described (McKenzie et al., 2014) with hemizygous littermates MyRF^+/−^ used as controls. The term MyRF^DHF^ denotes homozygous mice treated with 7,8-DHF. Forty-seven male and forty-four female mice were housed in groups of two to five under a 12 h light/dark cycle and were provided with *ad libitum* access to food and water. Behavioral training and testing were performed during the light phase at the same time each day. Increased stress responses to male experimenters have been observed in rodents (Faraji et al., 2022; Sorge et al., 2014). To counter any potential experimenter-induced side effects, experiments were undertaken by the same female experimenter throughout. Particular attention was also taken to use animal-handling techniques that reduce stress and promote animal welfare (Sensini et al., 2020).

### Morris water maze

The MWM is the most commonly used behavioral test of spatial memory (Morris, 1981). The mice are placed into a circular pool and learn to find a hidden platform that is submerged beneath the surface of opaque water. To do this, the mice need to use surrounding spatial cues. This behavioral test has been used in numerous previously published research articles and therefore has the advantage that performance can be compared between studies. Other advantages of this test include the uniformly motivating aspect of swimming in water, minimal training, limited subject dropout compared with many other learning paradigms, and no need for dietary food or water restriction (Vorhees and Williams, 2014). The main disadvantage of the MWM is that it can be stressful for the mice (Vorhees and Williams, 2014). However, given that the mice learn the task quickly, it is questionable as to how debilitating the stress caused is. It can also be argued that the prolonged food or water restriction required by alternative behavioral tasks is equally stressful. Steps were taken to limit stress caused to the mice in this experiment including using low-level lighting and limiting the number of trials to three on the first day of training when the mice were first exposed to the water. Experimenter-induced stress was also limited as outlined in the section above.

The MWM (diameter, 2 m) was filled with water to a depth of ∼0.29 m. The mice were required to find a hidden platform (diameter, 21 cm) with fixed location and submerged ∼1 cm below the water surface. The swim paths of the mice were recorded and tracked using WaterMaze software (Actimetrics). For the training period, the mice undertook three trials per day for the first day and then four trials per day for a total of 7 d. In each trial, they were placed into the pool at one of eight different starting points selected randomly. The mice had a maximum of 90 s to find the platform. Once found, the mice remained on the platform for 15 s. If the mice were unsuccessful in locating the platform within the 90 s, they were gently guided to it by the experimenter. The mice were placed into a warming box to dry off following all training and testing sessions. The mice underwent a probe test 28 d later. During the probe test, the platform was removed and the mice swam freely for 45 s. The percentage of time they spent in the four quadrants of the maze along with their average speed were recorded.

### Drug preparation and administration

Tamoxifen (300 mg/kg; Sigma-Aldrich) was administered at approximately postnatal day 70 by gavage for 4 d to induce the inactivation of *Myrf* in OPCs. Tamoxifen was prepared fresh on the day of administration by diluting it with corn oil (Sigma-Aldrich) to a concentration of 40 mg/ml as previously outlined (McKenzie et al., 2014). The mice were given at least 3 weeks to recover from any side effects of the tamoxifen, such as weight loss, before behavioral testing. 7,8-DHF (Sigma-Aldrich) was dissolved in 17% DMSO in PBS. MyRF^DHF^ mice received one intraperitoneal (IP) injection of 7,8-DHF 5 mg/kg, immediately following each MWM training session (seven in total), while MyRF^−/−^ and MyRF^+/−^ mice received intraperitoneal injections of vehicle (17% DMSO in PBS). Care was taken to administer IP injections on alternating sides of the abdomen to limit sensitivity. The dose of 7,8-DHF has been widely used and shown to improve symptoms in a number of disease models (Zeng et al., 2012; Zhang et al., 2014; Stagni et al., 2017) 5-Ethynyl-20-deoxyuridine (EdU; Sigma-Aldrich) was administered to the mice via drinking water at a concentration of 0.2 mg/ml for 4 d starting on the last day of training.

### Immunohistochemistry

On the day of the probe test, the mice were euthanised using an overdose of pentobarbital and perfused with 4% PFA at rate of 2 ml/min. The brains were postfixed for 24 h in 4% PFA and stored in 20% sucrose solution before embedding in O.C.T. (optimal cutting temperature) compound and coronal sectioning at 25–30 μm. For free-floating immunohistochemistry sections were blocked with 10% fetal bovine serum (FBS; Thermo Fisher Scientific) and 0.5% Triton X-100 (Sigma-Aldrich) in TBS at room temperature (RT) for 2 h. The sections were then incubated with primary antibodies mouse anti-adenomatous polyposis coli clone CC-1 (CC1; 1:200; catalog #OP-80, Calbiochem) and PDGF receptor α (1:500; catalog #3164S, Cell Signaling Technology) or anti-NeuN (1:1000; catalog #EPR12763 Abcam), in 5% FBS and 0.25% Triton X-100 in TBS at 4°C for 16 h. Following washing with TBS, the sections were incubated with secondary antibodies in 1% FBS and 0.1% Triton X-100 in TBS at RT for 1.5 h. The sections were then washed with 1× PBS followed by EdU staining for 30 min at RT. The following secondary antibodies and nuclei stains were used: goat anti-mouse Alexa Fluor 488 and donkey anti-rabbit Alexa Fluor 568 (both 1:500; Thermo Fisher Scientific); Click-iT EdU Alexa Fluor 647 (Thermo Fisher Scientific); Hoechst 33342 (1:1000; Thermo Fisher Scientific). Sections were mounted using Mowiol mounting medium. Confocal microscopy was performed with a microscope (catalog #FV1000, Olympus) equipped with Fluoview software. Three coronal slices were imaged at 20× or 40× magnification for each animal. The *z*-stack (10 steps, 1.3 μm) images were analyzed using Fiji software (www.imagej.net/Fiji). A region of interest with an area of 0.081 mm was used in the corpus callosum, and 0.38 mm for the dentate gyrus (DG). The number of EdU^+^/CC1^+^ and EdU^+^/Pdgfrα^+^ cells in the corpus callosum and the number of EdU^+^/NeuN^+^ cells in the DG were counted manually in each region of interest using the Fiji cell counter plugin.

### Statistical analysis

Statistical analyses were conducted using R studio (version 2021.09.2). Data were assessed for homogeneity of variances using Levene’s test and normal distribution using the Shapiro–Wilk test. Comparisons of groups were undertaken using one-way ANOVA, two-way mixed ANOVA, Kruskal–Wallis test, *t* tests, and Wilcoxon test. Least significant difference (LSD) *post hoc* tests were used. Data are presented as the mean ± SD with graphs generated in GraphPad Prism (version 9.3.0). Because of technical difficulties, the swim speed of two mice and the time to find the platform of one mouse during the training period were not recorded and therefore were not included in the analysis.

## Results

### Long-term spatial memory rescued in MyRF^−/−^ mice following 7,8-DHF administration

**Figure 1.**
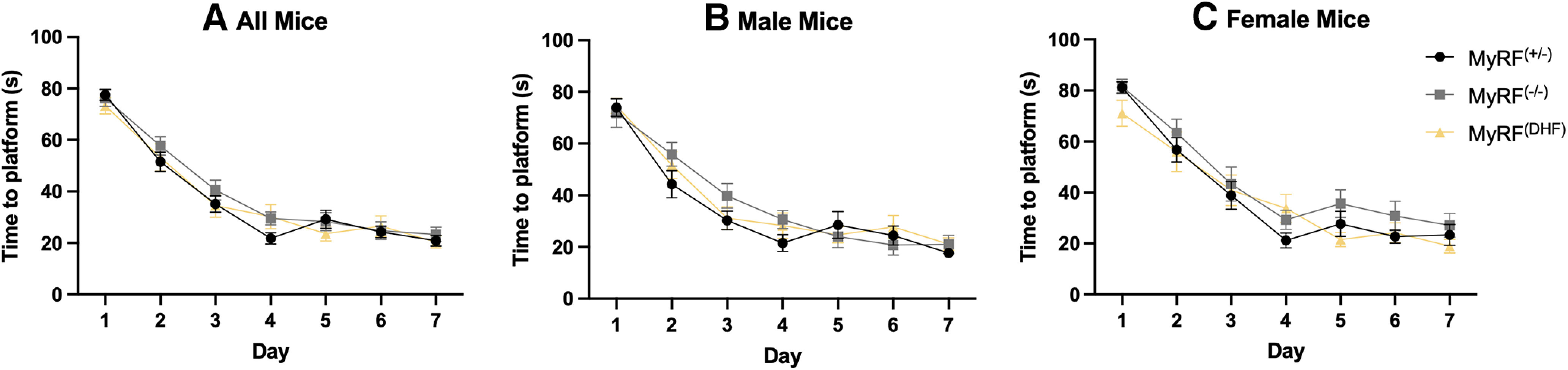
Performance of MyRF mice in the Morris water maze during the training period. ***A***, All mice took a similar amount of time to find the platform during the training period. ***B***, Time taken to find the platform for male mice. ***C***, Time taken to find the platform for female mice. Data are presented as the mean ± SEM.

All groups of MyRF^+/−^ [*n* = 29; male (m) = 16/female (f) = 13], MyRF^−/−^ (*n* = 23; m = 12/f = 11), and MyRF^DHF^ (*n* = 26; m = 17/f = 9) successfully undertook MWM training. Over the 7 d, the time taken to find the platform decreased, indicating successful spatial memory acquisition (two-way mixed ANOVA; *F*_(2,74)_ =193.5, *p* < 0.001; Fig. 1*A*). No difference was seen between groups (*F*_(2,74)_ = 0.619, *p* = 0.541; groups × day interaction: *F*_(2,74)_ = 1.2, *p* = 0.28). The speed of the mice increased during the 7 d (*F*_(2,72)_ = 3.948, *p* < 0.001), but there was no main effect of group (*F*_(2,72)_ = 0.699, *p* = 0.5) or interaction (*F*_(2,72)_ = 0.380, *p* = 0.970). The time taken to find the platform was also decreased in male mice (two-way mixed ANOVA; *F*_(2,41)_ = 98.91, *p* < 0.001; Fig. 1*B*) and female mice (two-way mixed ANOVA; *F*_(2,30)_ = 98.31, *p* < 0.001; Fig. 1*C*) when analyzed separately.

**Figure 2.**
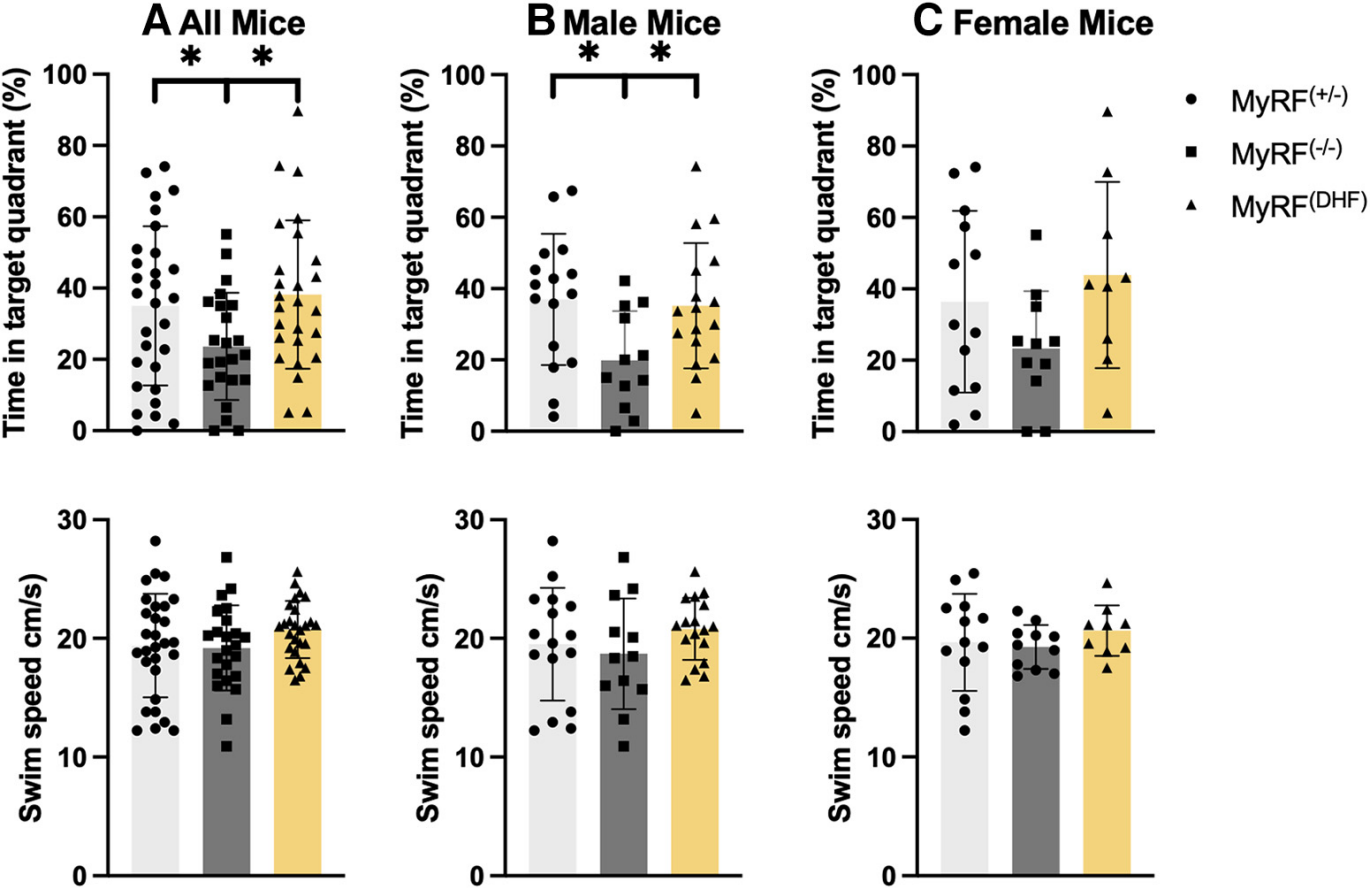
Performance of MyRF mice in the Morris water maze during the 28 d probe test. ***A***, The MyRF^−/−^ mice spent a smaller percentage of time in the target quadrant compared with the MyRF^+/−^ controls during the 28 d probe test. The MyRF^DHF^ mice that received 7,8-DHF immediately after each training session spent a larger percentage of time in the target quadrant compared with the MyRF^−/−^ mice, indicating rescue of long-term spatial memory by 7,8-DHF. The swim speed of the mice was comparable for all groups during the probe test. ***B***, Percentage of time in the target quadrant and swim speed for male mice. ***C***, Percentage of time in the target quadrant and swim speed for female mice. Data are presented as the mean ± SD. *p < 0.05.

**Figure 3.**
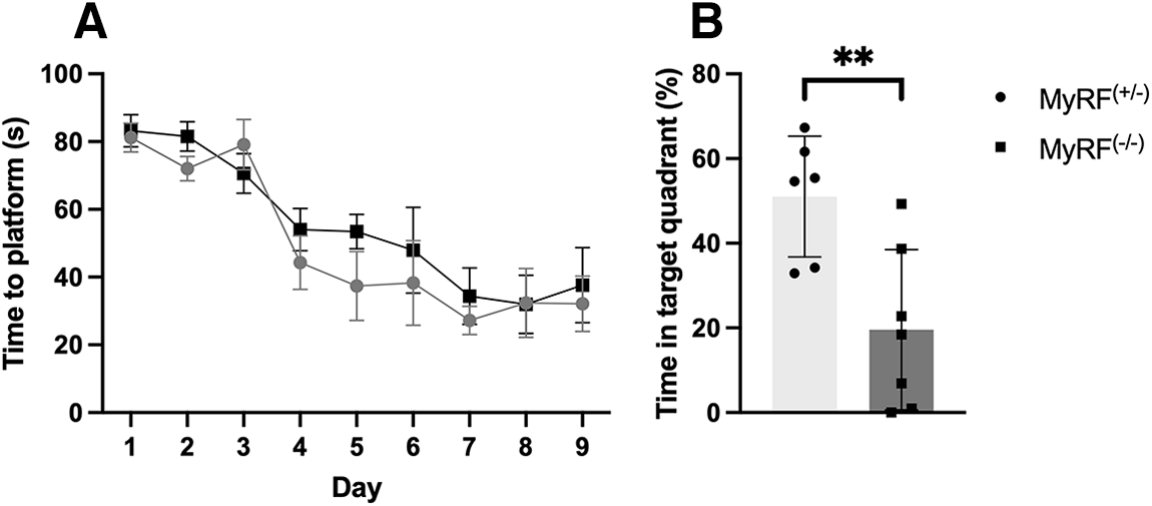
Spatial memory is impaired in MyRF^−/−^ female mice trained for 9 d. MyRF^+/−^ and MyRF^−/−^ female mice successfully undertook MWM training. ***A***, Time taken to find the platform decreased over the 9 d, indicating successful spatial memory acquisition in both groups. ***B***, During the 28 d probe test, the percentage of time spent in the target quadrant was lower in the MyRF^−/−^ compared with the MyRF^+/−^ female mice, indicating impaired long-term spatial memory. Data are presented as the mean ± SEM or SD. **p < 0.01.

Twenty-eight days after training, the mice underwent a 45 s probe test. The control mice and the mice that received 7,8-DHF spent a greater percentage of time in the target quadrant, where the platform had previously been located during training (MyRF^+/−^ median = 37.22%; MyRF^DHF^ Mdn = 35.53%) compared with the other quadrants (MyRF^+/−^ Mdn = 20.92%; MyRF^DHF^ Mdn = 21.48%), indicating intact spatial memory (Wilcoxon test; MyRF^+/−^: W = 123, *p* = 0.04; MyRF^DHF^: W = 65, *p* = 0.004). The MyRF^−/−^ mice did not show any preference for the target quadrant (Mdn = 21.26%) compared with the other (Mdn = 26.2%) quadrants (Wilcoxon test; W = 123, *p* = 0.659). Accordingly, the percentage of time spent in the target quadrant was lower in the MyRF^−/−^ mice compared with the MyRF^+/−^ and MyRF^DHF^ groups (one-way ANOVA: *F*_(2,75)_ = 3.559, *p* = 0.033; *post hoc* LSD: MyRF^+/−^ > MyRF^−/−^; MyRF^DHF^ > MyRF^−/−^; Fig. 2*A*). These results show that 7,8-DHF administered immediately following training sessions is able to rescue the impaired long-term spatial memory that occurs in MyRF^−/−^ mice 28 d later. MyRF^+/−^ and MyRF^DHF^ male mice spent a greater percentage of time in the target platform (MyRF^+/−^ Mdn = 39.85%; MyRF^DHF^ Mdn = 33.74%) compared with the other platforms (MyRF^+/−^ Mdn = 20.05%; MyRF^DHF^ Mdn = 22.08%; Wilcoxon test; MyRF^+/−^: W = 26, *p* = 0.02; MyRF^DHF^: W = 31, *p* = 0.03). This was not the case for the MyRF^−/−^ group, quadrant (Mdn = 17.53%) compared with the other quadrants (Mdn = 27.48%; Wilcoxon test; MyRF^−/−^: W = 24, *p* = 0.26). The percentage of time spent in the target quadrant was lower in the MyRF^−/−^ mice compared with the MyRF^+/−^ and MyRF^DHF^ groups (one-way ANOVA: *F*_(2,42)_ = 4.05, *p* = 0.02; *post hoc* LSD: MyRF^+/−^ > MyRF^−/−^; MyRF^DHF^ > MyRF^−/−^; Fig. 2*B*). MyRF^+/−^ and MyRF^DHF^ female mice spent more time in the target quadrant (MyRF^+/−^ Mdn = 29.95%; MyRF^DHF^ Mdn = 41.30%) compared with the other platforms (MyRF^+/−^ Mdn = 23.35%; MyRF^DHF^ Mdn = 19.56%). However, this did not reach a significance (Wilcoxon test; MyRF^+/−^: W = 24, *p* = 0.14; MyRF^DHF^: W = 8, *p* = 0.09), whereas the MyRF^−/−^ group spent a similar percentage of time in the target quadrant (Mdn = 24.64%) compared with the other quadrants (Mdn = 25.12%; Wilcoxon test; MyRF^−/−^: W = 29, *p* = 0.755). There was also a similar pattern to the male mice showing a lower percentage of time spent in the target quadrant by the MyRF^−/−^ mice compared with the MyRF^+/−^ and MyRF^DHF^ but again this did not reach significance (one-way ANOVA: *F*_(2,30)_ =1.189, *p* = 0.14; Fig. 2*C*). The lack of significant difference in female mice is hard to interpret. Data from an additional batch of female MyRF^−/−^ (*n* = 7) and MyRF^+/−^ (*n* = 6) mice tested in the same MWM but trained for 9 d rather than 7 d did show a significant group difference in the percentage of time spent in the target quadrant during the 28 d probe test (Student’s *t* test: *t* = 3.32, df = 11, *p* = 0.006; Fig. 3*B*). Both groups also successfully undertook spatial memory acquisition (two-way mixed ANOVA: *F*_(1,11)_ = 22.06, *p* < 0.001; Fig. 3*A*). It is possible that estrous cycle stage impacted outcomes in the female mice. Estrous cycle stage has been shown to impact spatial memory performance in female subjects (Frye et al., 2021; Patel et al., 2022). The average swim speed of the mice during the probe test was found to be comparable among the MyRF^+/−^, MyRF^−/−^, and MyRF^DHF^ groups (one-way ANOVA: *F*_(2,75)_ = 1.407, *p* = 0.251; Fig. 2*A*) . The average swim speed was also comparable among the three groups when males (one-way ANOVA: *F*_(2,42)_ = 1.00, *p* = 0.376; Fig. 2*B*) and females (one-way ANOVA: *F*_(2,30)_ = 0.54, *p* = 0.589; Fig. 2*C*) were analyzed separately.

### Increased number of new oligodendrocytes in the corpus callosum of MyRF^−/−^ mice following 7,8-DHF administration

**Figure 4.**
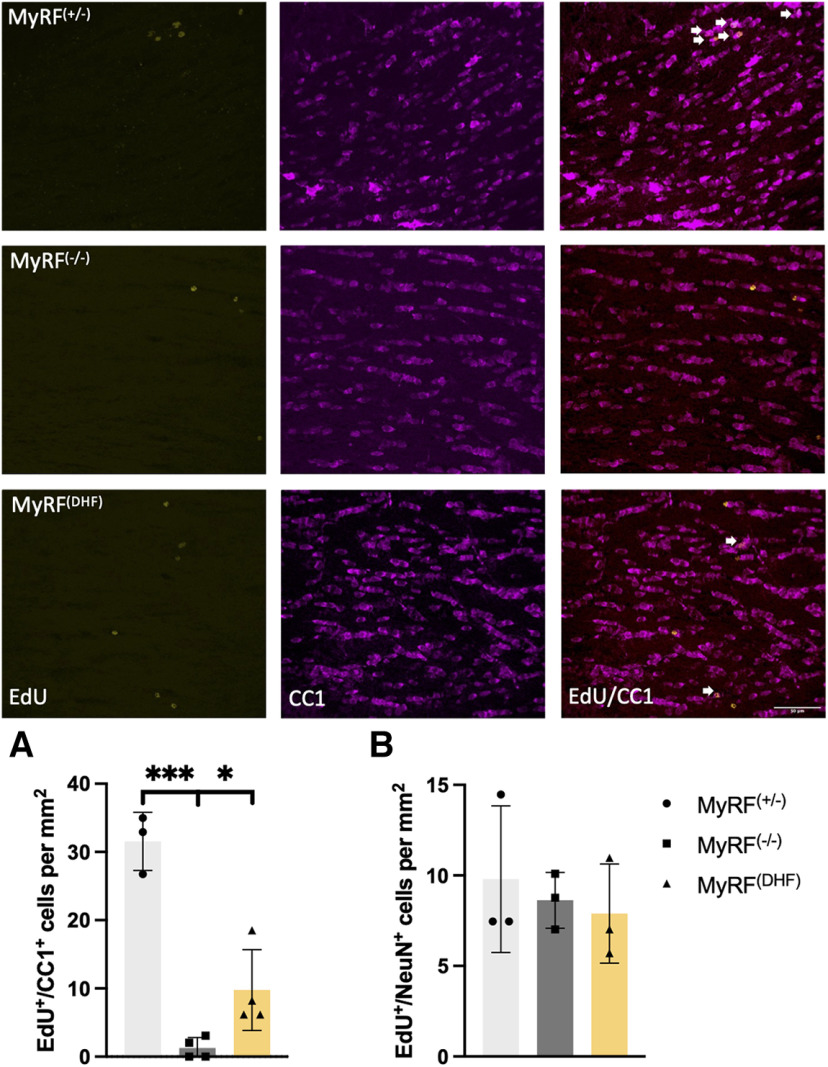
Increased numbers of new oligodendrocytes in the corpus callosum of MyRF^−/−^ mice following 7,8-DHF administration. Representative images of corpus callosum in MyRF^+/−^, MyRF^−/−^, and MyRF^DHF^ mice showing EdU^+^ cells in yellow, CC1^+^ cells in magenta, and a merged image. EdU^+^/CC1^+^ cells are indicated with arrows. ***A***, The number of EdU^+^/CC1^+^ cells was lower in the MyRF^−/−^ mice compared with the group that received 7,8-DHF and the MyRF^+/−^ control group. This indicates that the number of new mature oligodendrocytes was increased by administration of 7,8-DHF in the corpus callosum. ***B***, No difference was seen in the number of EdU^+^/NeuN^+^ cells in the dentate gyrus, indicating no change in the numbers of newly formed neurons. Data are presented as the mean ± SD. ***p < 0.001, *p < 0.05.

The number of EdU^+^/CC1^+^ cells in the corpus callosum varied across groups (one-way ANOVA; *F*_(2,8)_ = 43.63, *p* < 0.001). This reflected a reduction in the MyRF^−/−^ group (*n* = 4; m = 3/f = 1) group compared with the MyRF^+/−^ group (*n* = 3; m = 2/f = 1; *post hoc* LSD, MyRF^+/−^ > MyRF^−/−^; Fig. 4*A*) demonstrating that the MyRF^−/−^ mice had reduced numbers of newly formed oligodendrocytes in the corpus callosum following tamoxifen treatment, as expected. There was also a reduction in the MyRF^−/−^ group compared with the MyRF^DHF^ group (*n* = 4; m = 2/f = 2; *post hoc* LSD, MyRF^DHF^ > MyRF^−/−^; Fig. 4*A*), suggesting that 7,8-DHF administration during the training period increases the numbers of newly formed oligodendrocytes in the corpus callosum of MyRF^−/−^ mice, although numbers were not fully rescued to the same level as the MyRF^+/−^ controls (LSD MyRF^+/−^ > MyRF^DHF^). The number of EdU^+/^Pdgfrα^+^ cells was comparable between groups (one-way ANOVA: *F*_(2,8)_ = 1.847, *p* = 0.219), indicating that the number of new OPCs was not impacted.

### Number of newly formed neurons unchanged in the dentate gyrus of MyRF^−/−^ and MyRF^DHF^ mice

The number of EdU^+^/NeuN^+^ cells in the DG of the hippocampus did not vary significantly among the MyRF^−/−^ (*n* = 3; m = 2/f = 1), MyRF^+/−^ (*n* = 3; m = 2/f = 1), and MyRF^DHF^ (*n* = 3; m = 2/f = 1) groups (Kruskal–Wallis test; *H*(2) = 0.972, *p* = 0.615; Fig. 4*B*), indicating that adult neurogenesis in the hippocampus was not altered by *Myrf* KO or 7,8-DHF administration.

## Discussion

We found that long-term (28 d) spatial memory was impaired in mice with reduced oligodendrogenesis. MyRF^−/−^ mice spent a smaller percentage of time in the target quadrant of the MWM when compared with their sibling controls. This finding agrees with previous research (Steadman et al., 2020). We extended this work to show that 7,8-DHF administered immediately following each training session prevents this long-term memory impairment. 7,8-DHF administration was also found to increase the numbers of newly formed oligodendrocytes in the corpus callosum compared with vehicle-injected controls. This work indicates that 7,8-DHF rescues the spatial memory impairment found in mice with a conditional KO of *Myrf* in adulthood.

The numbers of newly formed oligodendrocytes were not shown to be fully restored in the MyRF^DHF^ compared with the numbers observed in the MyRF^+/−^ mice, but they were significantly increased compared with the MyRF^−/−^ mice. This suggests that the restoration of behavior was, at least in part, mediated by newly formed oligodendrocytes. The lack of full rescue of newly formed oligodendrocytes could have been a result of the experimental timing of EdU administration. In this experiment, EdU was administered for 4 d following the end of MWM training, therefore only sampling a small “time window” in the experimental procedure. It is possible that had EdU been administered for a longer time period or during a different stage of the protocol, the increased numbers of EdU^+^/CC1^+^ cells seen in the MyRF^DHF^ group would have been comparable to the numbers observed in the MyRF^+/−^ mice. It is also possible that there is redundancy in the system and that only a small number of newly formed oligodendrocytes are needed to mediate the full behavioral improvement observed in this study.

7,8-DHF is thought to mimic the action of BDNF (Jang et al., 2010; Massa et al., 2010). An association between myelination and BDNF is suggested by previous research using other TrKB agonists, including agonist tricyclic dimeric peptide 6 (TDP6) and LM22A-4. TDP6 enhanced myelination by oligodendrocytes *in vitro* (Wong et al., 2014), and both TDP6 and LM22A-4 improved remyelination in the cuprizone demyelination mouse model (Fletcher et al., 2018; Nguyen et al., 2019). Remyelination was also found to be enhanced in a Wolfram syndrome animal model following 7,8-DHF administration (Seppa et al., 2021). There is emerging evidence that the mitogen-activated protein kinase pathway, which leads to the activation of extracellular signal-related kinase 1 and 2 (ERK1/2) could have an important role in myelination (Gaesser and Fyffe-Maricich, 2016). BDNF has been shown to modulate intermediate kinase Fyn, which interacts with this pathway (Peckham et al., 2016). ERK1/2 was also found to be increased in the hippocampus of older rats chronically administered 7,8-DHF (Zeng et al., 2012). However, it has been suggested that 7,8-DHF may not be a BDNF agonist instead binding with high affinity to other receptors including adenosine receptor types 1 and 3, melatonin receptor type 3, and GABA_A_ receptor α1 benzodiazepine (Pankiewicz et al., 2021).

An alternative explanation for the rescue of long-term spatial memory by 7,8-DHF found in this study could be enhanced neurogenesis. However, we found that the number of newly formed EdU^+^/NeuN^+^ neurons in the dentate gyrus was comparable among groups, indicating that 7,8-DHF did not increase hippocampal adult neurogenesis in the MyRF^−/−^ mice. Previous research looking at the effect of 7,8-DHF on neurogenesis is mixed. Increased numbers of NeuN^+^ cells were found in the hippocampus of perimenopausal mice administered 7,8-DHF (Amin et al., 2020). Increased neurogenesis was also reported in juvenile Down syndrome mice administered 7,8-DHF (Stagni et al., 2017); however, this was not replicated in adult Down syndrome mice (Giacomini et al., 2019). Increased neurogenesis was also observed in the hippocampus of the AD mouse model APP/PS1 following 7,8-DHF administration (Hsiao et al., 2014), but not in the 5XFAD model (Aytan et al., 2018). Interestingly, increased neurogenesis was reported in the hippocampi of mice given the BDNF antagonist ANA-12 (Groves et al., 2019).

It is also possible that 7,8-DHF is restoring long-term spatial memory independent of *Myrf* and newly formed oligodendrocytes, for example by enhancing synaptic plasticity in isolation. BDNF has been shown to be important for long-term potentiation and synaptic plasticity (Kowiański et al., 2018; de Vincenti et al., 2019). However, to our knowledge, these studies did not control for the role of newly formed oligodendrocytes. Synaptic plasticity alone would appear to be insufficient for long-term spatial memory formation given the impairment observed in MyRF^−/−^ mice. We suspect that that both synaptic plasticity and oligodendrogenesis are required and work in synergy. Additional experiments that dissociate these two processes and assess how they are modulated by 7,8-DHF could be beneficial. It is likely that 7,8-DHF is exerting its effect on long-term memory by targeting mechanisms downstream of *Myrf*. For example, previous work found that dual-specificity phosphate 15 expression was reduced in the hippocampus of the MyRF^−/−^ mice (Rawlings-Mortimer et al., 2023). Future research to investigate the role of 7,8-DHF on molecular pathways associated with oligodendrogenesis and its impact on specific receptor types for example TrkB and GABA_A_ would be valuable.

In conclusion, we found that 7,8-DHF improves the long-term spatial memory of mice with reduced adult oligodendrogenesis. 7,8-DHF has previously been shown to improve the spatial memory in a number of mouse models of disease, including AD, PTSD, Wolfram syndrome and Down syndrome. This work helps to shed light on potential mechanisms underlying these outcomes, namely oligodendrogenesis. This could therefore help in the assessment of 7,8-DHF or the development of similar drugs for future clinical use.

## Synthesis

Reviewing Editor: Erica Glasper, The Ohio State University Decisions are customarily a result of the Reviewing Editor and the peer reviewers coming together and discussing their recommendations until a consensus is reached. When revisions are invited, a fact-based synthesis statement explaining their decision and outlining what is needed to prepare a revision will be listed below. The following reviewer(s) agreed to reveal their identity: NONE.

Synthesis: In this manuscript, the authors report that deletion (or reduction) of oligodendrocytes via conditional KO (of myelin regulatory factor (Myrf) in oligodendrocyte precursor cells (OPCs) is associated with a reduction in long term spatial memory in adult mice. In addition, treatment the TrkB agonist 7,8-DHF, post-training reduces long-term memory impairment and increases the numbers of oligodendroglia in conditional KO mice. The reviewers find the results novel and of interest to the field. The manuscript is well-written, and the illustrations rightly present the main findings of the study. The paper describes an interesting effect of the myelin regulatory factor conditional knockouts on spatial memory and describes that this deficiency is offset by 7,8-DHF treatment. However, the data do not show 7,8-DHF effects on the numbers of newly generated oligodendroglia mediate the rescue of retention. This later point refers to major concerns (minor ones exist also) with experimental design that must be addressed. When appropriate, a specific reviewer comments have been quoted.

Major Concerns

1. The authors should include Myrf-/+ mice treated with 7,8-DHF. Without this group, it is not known if 7,8 DHF influences long term memory in all mice (controls and cKOs). This would inform the reader as to whether 7,8 DHF has a stand-alone effect or if 7,8 DHF is restoring something specific that is lost or disturbed in the conditional knockout. This group is required so that the results can be fully interpretable

2. “A second concern pertains to the absence of considering a very likely effects of 7,8-DHF treatment that would indeed be expected to enhance long term memory. To appreciate this issue one needs to understand that although mixed targets of DHF action have been described (as appropriately noted by the authors), the compound does increase levels of phosphorylated (activated) TrkB, an effect consistent with the putative role of this compound as a mimic for BDNF or, more appropriately termed, a TrkB agonist. Spatial memory is at least in part dependent on hippocampus, BDNF/TrkB signaling is required for synaptic plasticity underlying forms of hippocampus-dependent spatial learning, and 7,8-DHF has been described to facilitate hippocampus dependent learning in animals with deficiencies. Moreover the effect of BDNF/TrkB on plasticity in hippocampus is on stabilization of changes in synaptic function (in electrophysiological studies BDNF stabilizes synaptic potentiation and thus acts after the period of increased drive to consolidate plasticity and make it endure). There is thus a good amount of evidence that increasing BDNF levels, or increasing TrkB activity, will facilitate synaptic plasticity and learning through direct effects on synaptic changes that mediate encoding. This background information (all from published works) does not discount the possibility that newly identified functions of oligodendroglia may also be required for spatial learning, or that elevated TrkB activity might enhance the contributions of the oligodendroglia. However this background does provide another interpretation for how 7,8 DHF could facilitate long term spatial memory if the compound is supplied immediately after training (as was the case in the current experimental design); i.e., the DHF could be enhancing stabilization of synaptic plasticity needed for long term memory. Minimally this route of action should be considered. Beyond this, to further explore potential oligodendroglial involvement, one could possibly dissociate effects of the compound on synaptic consolidation and on oligodendroglia by providing the compound by a different regimen and verifying that treatments do not increase BDNF levels. Thus, 7,8 DHF might be given the night before training so that levels would not be elevated during training or soon thereafter: Such a regiment might still increase numbers of newly formed oligodendroglia without having effect on long term memory (but for this idea one would need to assess BDNF levels within hippocampus which, if elevated, could bridge the 7,8 DHF treatments and influences consolidation).”

Minor Concerns

1. Please define the MyrfDHF group in the methods section. If appropriate, please explicitly state 7,8-DHF treatment was restricted to the conditional knockouts.

2. Please include labeling within the figure when identifying the panels. For example, figure 1 could give a heading for panel B that would indicate male results and panel C would indicate females results.

3. Please brighten the images in the right column of figure 3, as it is difficult to see the yellow fluorescent cells in the left column.

4. Please refrain from referring to comparisons that did not reach statistical significance as a “trend”. It is appropriate to state that one group has values that are higher than another but describing findings as borderline or ‘close’ effects should be carefully handled.

5. Since the authors report a sex difference in Morris water maze behavior, the authors should report estrous cycle stage and discuss how estradiol levels may influence spatial learning and may have prevented an observation of significant differences in females (even though a similar pattern was observed as shown in males).
